# Self-Flagellation as Possible Route of Human T-Cell Lymphotropic Virus Type-1 Transmission

**DOI:** 10.3201/eid2504.180984

**Published:** 2019-04

**Authors:** Alice R. Tang, Graham P. Taylor, Divya Dhasmana

**Affiliations:** Imperial College London, London, UK (A.R. Tang, G.P. Taylor);; St. Mary’s Hospital, London (G.P. Taylor, D. Dhasmana)

**Keywords:** self-flagellation, bloodborne viruses, transmission, human T-cell lymphotropic virus, HTLV-1, viruses, HIV/AIDS and other retroviruses, United Kingdom

## Abstract

We report human T-cell lymphotropic virus type 1 infection associated with self-flagellation in 10 UK residents. These persons were heterosexual men from Pakistan, India, and Iraq. One person showed seroconversion in adulthood; 1 was co-infected with hepatitis C virus. No other risk factors for bloodborne virus acquisition were identified. Onward sexual transmission has occurred.

Human T-cell lymphotropic virus type 1 (HTLV-1) is transmitted sexually, by contaminated blood products, by organ transplantation, or from mother to child. The estimate of 5–10 million global infections excludes 85% of the general population, for which testing has not occurred, and is probably an underestimate (*1*). Disease occurs in <10% of carriers. In 2%–6%, adult T-cell leukemia/lymphoma develops; this condition has a high mortality rate and a median survival of 8–10 months despite therapy (*2*). In 0.25%–3.8 %, HTLV-1–associated myelopathy develops; this condition has a high morbidity rate, and many other inflammatory conditions have been reported (*3*).

Self-flagellation, one of several practices in which piercing of the body occurs as part of religious practice, typically involves beating the back with implements attached to ropes or chains, resulting in skin lacerations, as part of a public or private religious practice. The implements might be knives or blades, as used by the Pakistani Shia community, in which the practice is referred to as zanjeer, or may involve whips or rods. Alternatively, in tatbir, practiced predominantly by Shia communities in the Middle East, the forehead is struck with a knife.

Self-flagellation has been practiced throughout history by different religious groups, usually only by men. It is a controversial practice, even among some of the Shia Islamic and Catholic communities that continue it. It occurs worldwide but notably in Iraq, Lebanon, Afghanistan, and India. Self-flagellation has also been documented to occur in Catholic communities (*4*). Although self-flagellation is widely reported by the media, there are no statistics regarding its prevalence. We report HTLV-1 infection associated with self-flagellation in 10 UK residents.

## The Study

Case-patient A was given a diagnosis of HTLV-1 infection during screening before he and his wife undertook in vitro fertilization (IVF). The patient was of Indian origin and had lived in the United Kingdom since early adulthood. He provided no history of receiving blood products, tattoos, or injection drug use. No family history was suggestive of HTLV-1 infection. In 2008, he donated blood in the United Kingdom that was tested for HTLV-1; he was seronegative. His wife of 10 years was also negative for HTLV-1. He had engaged in zanjeer voluntarily during childhood outside the United Kingdom and continued this practice. In the United Kingdom, the blades were soaked in a bucket containing an over-the-counter antiseptic solution, along with the blades of other men conducting the practice simultaneously. In the previous few years, his practice had also involved striking his forehead with a knife, which was subsequently shared by other men. Physical examination showed widespread scarring on his back (Figure) and the superior aspect of his scalp associated with self-flagellation.

**Figure:**
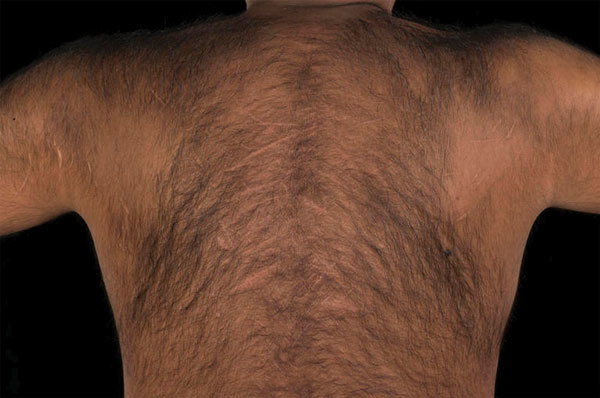
Back of case-patient A showing scarring from self-flagellation, United Kingdom.

We provide epidemiologic findings and HTLV-1 proviral load (HTLV-1 DNA copies/100 peripheral blood mononuclear cells) for this case-patient and 9 other asymptomatic HTLV-1 carriers of similar demography who reported a history of self-flagellation in Iraq, Pakistan, India, or the United Kingdom (Table). Most reported sharing of blades. Some had required sutures abroad. Eight patients had single lifetime sexual partners and no other risk factors for acquisition of bloodborne viruses. One man was co-infected with hepatitis C virus (HCV); all others were negative for HIV, hepatitis B virus, and HCV.

**Table:** Characteristics of 10 case-patients infected with HTLV-1 who practice self-flagellation, United Kingdom*

Case-patient	Age at diagnosis, y	Country of birth or ethnicity	HTLV-1 proviral load %	Route of diagnosis	HTLV-1 status of regular sexual partner	Sharing of equipment	Characteristic
A	34	Indian	0.8	Screening for IVF	Negative	Yes	Blood donor in UK 9 y earlier, documented HTLV negative
B	40	Pakistan	0.7	Cord blood donor (partner)	Positive	Yes	Hepatitis C virus co-infection now cured
C	47	Pakistan	0.8	Screening for IVF	Negative	Yes	Multiple previous blood donations in Pakistan
D	25	Pakistan	0.79	UK blood donor	No current partner	Yes	Previous blood donor in Pakistan
E	37	Pakistan	2.11	Cord blood donor (partner)	Positive	Yes	None
F	31	UK, Indian	0.14	UK blood donor	Negative	Yes	None
G	22	UK, Pakistani	0.4	UK blood donor	Positive	No	Received sutures in Iraq; wife seroconverted and became pregnant
H	33	UK, Indian	2.69	UK blood donor	Negative	Yes	None
I	37	Pakistan	Undetectable	UK blood donor	Negative	No	Sample/cutoff ratio >80†
J	38	Iraq	0.001	UK blood donor	Unknown	Yes	None

*All case-patients were male. HTLV-1, human T-cell lymphotropic virus type 1; IVF, in vitro fertilization. †For an enzyme immunoassay assay.

All 10 patients were given a diagnosis of infection with HTLV-1 through screening programs since 2013. Nine patients had strongly positive Western blot results and positive PCR results. One patient had indeterminate Western blot results and negative PCR results but an HTLV-1/2 enzyme immunoassay sample/cutoff ratio >80, which is consistent with HTLV infection (*5*). HTLV-1 proviral load is routinely measured in our center to monitor HTLV-1–infected carriers. Eight of the 10 patients had a low HTLV proviral load (<1%), which is typical of asymptomatic HTLV-1 infection and suggests a low risk for development of HTLV-1–associated disease. Two of the men had an HTLV-1 proviral load >1%, which is associated with a higher risk for complications.

## Conclusions

We describe 10 cases of HTLV-1 infection in men in whom the practice of self-flagellation was the only identifiable risk factor. In 1 patient, co-infection with HCV was also found. Mother-to-child transmission is difficult to exclude without testing all mothers of the case-patient, not all of whom are in the United Kingdom. However, the 1 screened mother was seronegative for HTLV-1, and case-patient A was uninfected when he donated blood 9 years earlier, which excludes maternal transmission. It is likely that either sharing blood-stained blades, reusing personal equipment after inadequate cleaning with a shared disinfectant, contact of infected blood with open wounds, or contact with infected medical equipment resulted in HTLV-1 transmission.

Self-flagellation has also been noted to result in pneumothorax (*6*). In addition, Ashura, the period during which it is practiced, has been associated with increased medically reported injuries (*7*).

The contribution of self-flagellation to the transmission of bloodborne viruses is unknown. In the United Kingdom, clinics that screen for these viruses (antenatal and sexual health settings) do not ask about the practice and do not screen for HTLV. We propose that self-flagellation be added to the list of risk factors that result in testing for bloodborne viruses, including HTLV-1. Blood transfusion services might screen for this practice when assessing potential blood donors; for 6 men in this study, blood donation was the route of diagnosis. However, screening blood donors for HTLV-1 infection is not universal. Absence of screening, particularly in regions where self-flagellation is practiced, could accelerate dissemination of this infection. The seroprevalence of HTLV in Pakistan is unknown, but a recent study reported a prevalence of 0.19% among low-risk blood donors (*8*).

Four men were given diagnoses of infection with HTLV-1 as a result of Human Fertilization and Embryology Authority licensing regulations (*9*) on the basis of a 2015 European Union directive. The directive requires facilities storing and processing human reproductive tissue to test for HTLV-1 in persons from areas of high prevalence, or with partners or parents from areas of high prevalence; that is, where infection is present in >1% of the general population or >1 case/10,000 persons for first-time blood donors (*10*).

Of the 7 couples for whom each partner’s diagnosis was known, 4 couples were serodiscordant, despite unprotected sex over many years. Sexual transmission has been associated with proviral load and duration of the relationship (*11*). One study reported an ≈1% per year risk for infection among serodiscordant couples (*12*), although further data are needed to quantify sexual transmission risk in new relationships. During the 4 years since the first of these 10 cases was diagnosed, there has been 1 case of sexual transmission (case-patient G) (Table), and the affected woman is in her first pregnancy. She had negative results for HTLV-1 on 2 previous occasions.

All 10 men had asymptomatic HTLV-1 infections, but 2 men had a high proviral load (>1%), which places them at risk for HTLV-associated disease. In all but 1 case-patient, HTLV-1 was the only bloodborne virus detected. Within heterosexual populations, transmission of HCV is commonly associated with using contaminated injection equipment. HTLV-1 predominance in our cohort suggests the presence of a pool of monoinfected persons and spread within this international community.

All patients have been advised by medical practitioners not to share implements during self-flagellation and to encourage fellow practitioners of flagellation to be tested for bloodborne viruses. Our visits to communities in which these practices occur to discuss risk elimination, raise awareness, and promote testing facilities have been favorably received, and risk reduction has been implemented.
